# Acknowledgement to Reviewers of *Cells* in 2017

**DOI:** 10.3390/cells7010006

**Published:** 2018-01-10

**Authors:** Cells Editorial Office

**Affiliations:** MDPI AG, St. Alban-Anlage 66, 4052 Basel, Switzerland

Peer review is an essential part in the publication process, ensuring that *Cells* maintains high quality standards for its published papers. In 2017, a total of 48 papers were published in the journal. Thanks to the cooperation of our reviewers, the median time to first decision was 19 days and the median time to publication was 39 days. The editors would like to express their sincere gratitude to the following reviewers for their time and dedication in 2017:

Aberle, Judith H.Heit, BryanOlins, DonaldAdam, Stephen A.Hirsch, PierreOsman, MohamedAlloza, IraideHolnthoner, WolfgangPalitti, FabrizioAn, Shi-QiIbeanu, GordonPellicioli, AchilleArndt, Patrick G.Iijima, MasahiroPetit, Patrice X.Arnhold, JuergenIngram-Smith, CherylPon, Liza A.Arumugam, SomasundaramJacoby, MeaganPossik, EliteBaird, NicholasJiang, LiwenProikas-Cezanne, TassulaBarbieri, FedericaKale, AbhijitPuckelwartz, Megan J.Berezowska, SabinaKanki, TomotakePugsley, HaleyBerkhout, BenKarlsson, ErikRamanathan, SudarshiniBerrett, Andrew N.Kim, Jeong-RaeRamani, KomalBertrand, Anne T.Klimaschewski, LarsRatajewski, MarcinBhutoria, SavitaKobayashi, SatoruRebelo, SandraBlasiak, JanuszKononenko, NataliaRout, BhimsenBosse, Jens B.Kortholt, ArjanRychkov, GrigoriBoya, PatriciaKropp, MartinaSaba, Nakhle S.Bruschi, FabrizioKuerten, StefanieSaera-Vila, AlfonsoBugliani, MarcoKurdowska, Anna K.Schlieker, ChristianBurger, MichaelLatif, NajmaSeiler, Magdalene J.Capitanio, NazzarenoLazarević, VladimirSewry, CarolineCarvajal, Agustin SolaLee, GabsangSgarra, RiccardoCastillo, KarenLeo, Chen HueiShachar, IditChang, Ko-TungLi, Shao-GangSilva, Jorge CarvalhoChartrand, PascalLiehn, Elisa A.Siqueira, Rubens C.Chiara, LanzuoloLimon, AgenorSlukvin, Igor I.Choulier, LaurenceLin, GialihSolovei, IrinaChueh, Pin JuLin, Pei-HuiSoong, Tuck WahCoirault, CatherineLoré, KarinStacchiotti, AlessandraCote, PatriceLow, PeterStewart, JasonDacheux, Jean-LouisLu, ZhanpingStorrie, BrianDaigaku, YasukazuLuo, WeiboSugahara, TakuyaDavis, Michael E.Mahfouz, RedaSugimoto, NaotoshiDe Meyer, GuidoMarkkanen, EnniTakats, SzabolcsDe Pater, SylviaMartini, MaurizioTakeshita, HaruoDeleidi, MichelaMcCall, KimberlyTanaka, TakashiD’elios, Mario M.McConkey, Glenn A.Temesvari, LeslyDing, Wen-XingMeinke, PeterThilmony, RogerDoddapaneni, RaviMinucci, SergioUchida, ShizukaDuan, ZhijunMisteli, TomUchiyama, YasuoDubielecka, Patrycja M.Miyagawa-Tomita, SachikoUnderwood, Mark D.Ebrahimi-Fakhari, DariusMohamed, Salah A.Vindis, CécileEngedal, NikolaiMonni, OutiVon Knethen, AndreasEstaras, ConcepcionMorgan, Michael A. M.Wang, ZhixiangEuler, GerhildMorishita, HideakiWong, Chung F.Everts, BartMoro, LoredanaXia, YuchenFoisner, RolandMuchir, AntoineXu, PengfeiForrester, Lesley M.Murdoch, Michele E.Xu, MiaoGan, BoyiMurotomi, KazutoshiYan, ShanGiannakouros, ThomasNahrendorf, WiebkeYao, BingGonzález-Maeso, JavierNajimi, MustaphaZhang, XuGoode, Bruce L.Nawaz, MuhammadZhang, Xiao-BoGuha, SoniaNissapatorn, VeeranootZhang, ChristieGundersen, Gregg G.Noda, TakeshiZhang, YuanHafizi, SassanObermair, Gerald J.
Heath, Joan K.Ohsumi, Yoshinori


